# Aging Independently of the Hormonal Status Changes Pain Responses in Young Postmenopausal Women

**DOI:** 10.1155/2012/693912

**Published:** 2011-10-03

**Authors:** Yannick Tousignant-Laflamme, Serge Marchand

**Affiliations:** ^1^School of Rehabilitation, Faculty of Medicine and Health Sciences, Université de Sherbrooke, Sherbrooke, QC, Canada J1H 5N4; ^2^Centre de Recherche Clinique Étienne-Le Bel du CHUS, Université de Sherbrooke, Sherbrooke, QC, Canada J1H 5N4; ^3^Neurosurgery, Faculty of Medicine and Health Sciences, Université de Sherbrooke, Sherbrooke, QC, Canada J1H 5N4

## Abstract

Both aging and hormonal status have an effect on pain perception. The goal of this study was to isolate as much as possible the effect of aging in postmenopausal women. Thirty-two women with regular menstrual cycles (RMW) and 18 postmenopausal women (PMW) underwent a 2-minute cold pressor test (CPT) to activate DNIC with a series of tonic heat pain stimulations with a contact thermode to assess ascending pain pathways. We found that this procedure induced much less pain during the first 15 seconds of stimulation the PMW group (*P* = 0.03), while the mean thermode pain ratings, pain tolerance, pain threshold, and DNIC analgesia were similar for both groups (*P* > 0.05). The absence of the peak pain in the PMW was probably due to reduced function of the myelinated A*δ* fibers that naturally occurs with age.

## 1. Introduction

Aging brings a decline in the majority of sensory modalities including pain and touch, two sensory modalities involving A and C fibers [1]. These two types of primary afferent fibers carry touch-(A-*β*) and pain-related information (A-*δ* and C). Although the observed decline in function of the sensory system is observed above 65 years of age, there is evidence that pain-related functions start to decline around middle age (*≈*50 years old) [2]. The results obtained by Larivière et al. showed a decline of function of the endogenous pain inhibitory system in middle age adults; this decline was similar to adults above 65 years old. Although these changes in pain perception can be due to aging, sex hormones could also be a factor influencing these changes since women reach menopause around 50 years of age and sex hormones levels significantly decrease. Although many biopsychosocial factors influence pain perception, it has been shown that sex hormones can influence many aspects of nociception [3]. This leads to the question: what happens to pain perception as women reach menopause?

Menopause, defined by the absence of menses for more than 12 months, usually occurs at about 50 years of age and results in significant changes in sex hormones levels: decreased progesterone (PRO) and estrogen (EST), increased luteinizing hormone (LH) and follicle-stimulating hormone (FSH). As we age, we generally tend to suffer from more pain-related pathologies [4], which are often more prevalent in women [5]. Comparable findings can be seen in midlife where postmenopausal women (PMW) are showing significant increases in clinical pain symptoms [6, 7]. Consequently, the changes in pain perception in this population of midlife women could be due to the decrease of sex hormones and/or aging. 

We found no previous studies that examined specific aspects of nociception and pain perception in PMW compared to women with a regular menstrual cycle (RMW). Fillingim and Edwards [8] studied the effect of hormonal replacement therapy (HRT) on PMW and found that women on HRT had lower pain thresholds than PMW not on HRT. Although these results conflict with what is usually found in young healthy women, they do seem to indicate that sex hormones could influence pain perception in an older population [8]. 

In our study, we used a cross-sectional design to examine pain perception in young PMW in comparison to RMW. Therefore, the study's main goal was to verify the influence of age on the ascending and descending pain mechanisms, while controlling for sex hormones levels, a potential confounding factor. To our knowledge, this has never been done and has significant clinical relevance for a large proportion of the population.

## 2. Methods

### 2.1. Participants

After approval from the hospital ethics review board, we collected data from 18 PMW (mean age 54.5 ± 5.4 years) and 32 RMW (mean age 34.3 ± 7.5 years). Subjects were recruited via local publicity and were all French-speaking women dwelling in the community. Brief initial phone interviews allowed for the screening of potential subjects and scheduling them for testing. On the day of testing, subjects were asked to refrain from smoking (only four RMW and three PMW self-reported as smokers) and/or drinking coffee one hour before testing. PMW were included if they met the Society of Obstetricians and Gynaecologists of Canada criteria for menopause (absence of menses during the past 12 months). Inclusion criteria for women in the RMW group were to have a regular menstrual cycle, which was defined as varying from 26 to 30 days in length. This criterion was confirmed by verifying menstrual-cycle length in the month before and after testing (self-reported). The average length was 28.4 ± 0.8 days. None of the RMW had any known disease or self-reported hypo/hypertension, or was taking pain medication. However, ten of the PMW reported having intermittent low back pain, but none were taking prescribed medication for this condition. Only three of them reported taking over-the-counter ibuprofen/acetaminophen as needed; when present, the mean intensity of their low back pain was 4/10 (numerical pain rating score).

All subjects signed an informed consent form and received $40 as compensation for taking part in the study. Each experimental procedure lasted about 90 minutes and took place at the *Centre de Recherche Clinique Étienne-LeBel du Centre Hospitalier Universitaire de Sherbrooke*, Sherbrooke, Québec, Canada.

### 2.2. Experimental Design

To control for a potential effect of sex hormones on pain responses, all RMW were tested during days 1 to 3 of their menstrual cycles, while PMW were tested at their convenience. We chose this time frame since sex hormones levels are at their lowest during menses. This enabled us to compare pain perception, while both groups have comparable sex hormones levels. The first day of menses was considered as day 1 of the menstrual cycle, which was obtained by self-reporting and confirmed by blood sampling, where we observed low levels of PRO, EST, and LH (all levels where within the normal reference values for this phase of the menstrual cycle). A qualified registered nurse took blood samples for 17*β*-estradiol, PRO, FSH, LH, and testosterone dosage, prior to each experimental session.

### 2.3. Pain Procedures

All subjects underwent the experimental procedures in the same order (experimental heat pain, cold-pressor conditioning stimulus, and experimental heat pain).

#### 2.3.1. Apparatus

The experimental heat pain was induced by a 9 cm^2^ thermode (TSA II, NeuroSensory Analyzer, Medoc Instruments, North Carolina, USA). During this stimulus, pain perception was assessed with a computerized visual analogue scale (COVAS) linked to the thermode, which was graduated from 0 (absolutely no pain) to 100 (maximum tolerated pain). This allowed us to determine pain threshold (PTh), as measured by thermode temperature at which subjects reported initial pain sensation (visual analogue scale score: 1/100); pain tolerance (PTol), as measured by the maximum thermode temperature subjects could tolerate (visual analogue scale score: 100/100); and mean pain intensity of the noxious tonic stimulus. The conditioning stimulus was induced by a cold pressor test (CPT), which consisted of immersing the right arm (up to the elbow) in circulating cold water maintained at 12°C. During the CPT, subjects were asked to rate their pain intensity every 15 seconds with a numerical pain rating scale ranging from 0 to 100.

#### 2.3.2. Pretest

Subjects were given a pretest for practicing pain rating with the visual analogue scale and to determine the temperature to be used for the heat pain test. The pretest was performed with the thermode applied to the right palm. For familiarization purposes, subjects were advised that the thermode temperature would gradually increase from 32°C to a maximum of 51°C (rising rate = 0.3°C/second). This procedure was repeated twice and the subjects verbally reported the point at which they actually began feeling pain (PTh) as well as PTol. On the third test, the thermode was placed on the volar aspect of the right forearm. Subjects were given the visual analogue scale and advised that they would have to start moving the cursor towards the right (towards the “100” mark) when they started to feel pain (PTh) and that the cursor had to be at the extreme right (at the “100” mark) when pain was intolerable (PTol) [9]. This procedure was repeated until the subject's pain reports were consistent between trials. The temperature used during the tonic experimental heat pain test was the temperature that the subject had rated pain intensity at 50/100 with the visual analogue scale during the pretest.

#### 2.3.3. Tonic Experimental Heat Pain Stimulus

**Figure 1:**
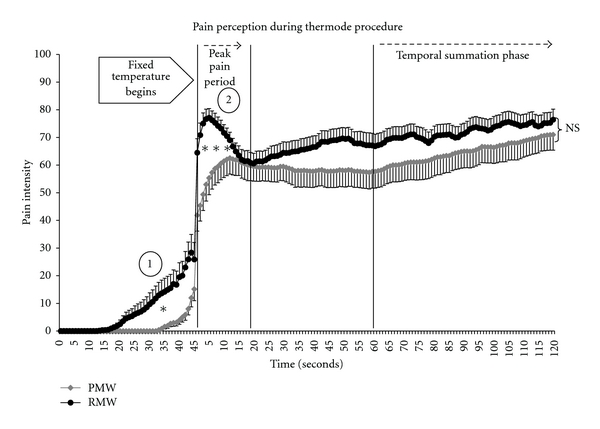
Average heat pain (thermode) intensity for all subjects during the “before” session (mean ± SE). (1) The PMW group took longer to report initial pain and (2) the RMW had much higher peak pain than the PMW. Finally, the temporal summation phase (last minute of stimulation) was similar for both groups.

The tonic heat pain test was performed by applying the thermode at a constant temperature to the anterior (volar) aspect of the left forearm for two minutes [10]. Before the procedure, subjects were told that the thermode temperature could increase, remain stable, or decrease, and that they would have to evaluate their pain with the visual analogue scale throughout the test. In fact, after a constant rise (0.3°C/second) from the baseline (32°C) to the predetermined temperature, the thermode temperature remained constant (mean = 46.1 ± 1.64°C—see Table 2 for mean thermode temperature used for each group) throughout the 120 seconds (ramp and hold). All subjects were blinded to the temperature used and to the study's hypothesis. Two observable events occurred during the tonic heat pain test [11, 12]. The first nociceptive event was characterized by a sharp but brief increase in pain intensity. This peak in pain intensity occurred when the thermode has reached its fixed temperature and lasted approximately 15 seconds. It was labeled “peak pain” because this is the interval in which heat sensitive A-delta fiber nociceptors display peak neuronal activity following constant stimulation at suprathreshold levels [13]. Peak pain has previously been described by Jensen and Petersen [14] using a similar design and repeated in our laboratories [9]. The second observable event was the rise in pain intensity that occurs during the last minute of stimulation. Since this increase in pain rating occurred at a set temperature, it clearly describes a temporal summation phenomenon (see Figure 1), which is known to depend on the summation of nociceptive inputs from primary afferent C-fibers. Granot et al. [15] also observed temporal summation effects using similar tonic heat stimulations. Previous research in our laboratory has shown that pain perception scores increase progressively during this tonic heat test, even if the temperature remains constant [9].

#### 2.3.4. Activation of Diffuse Noxious Inhibitory Controls

The conditioning stimulus (to induce DNIC) was applied to the opposite arm using the 12°C CPT. Pain intensity ratings were measured every 15 seconds during the test. If a subject removed her arm before the end of the 2 minutes, a pain intensity score of 100/100 was noted [16, 17]. The CPT enabled us to activate DNIC and was also used as a different type of tonic pain (cold pain versus heat pain; greater surface area than the 9 cm^2^ thermode).

#### 2.3.5. Assessment of DNIC Pain Modulation (Analgesia)

In order to measure the analgesic effect of the DNIC activated by the CPT, the experimental heat pain procedure was performed immediately after the immersion test using the same parameters. The amount of pain modulation produced by the CPT (DNIC efficacy) was calculated as the difference in pain score between the mean heat pain after and before the CPT. A negative score indicated a reduction in pain perception and therefore analgesia.

### 2.4. Statistical Analysis

Descriptive statistics are presented as means and standard deviations (SD) in the text and as mean and standard error in the figures. Since our data were normally distributed, a Student's *t*-test was used for group comparisons of pain perception measurements and to assess the presence of DNIC analgesia, comparing the average pain score between the first and second experimental heat pain tests (thermode procedure). Afterwards, we compared the difference score (mean pain after minus mean pain before) for both groups, which also allowed us to quantify DNIC analgesia. We used a *t*-test comparing the first and the last pain ratings during the last minute of the thermal stimulation with the thermode at constant temperature (*T*_60_ versus *T*_120_) in order to confirm the presence of temporal summation. Temporal summation was then quantified by calculating the mean difference score between the first and the last pain ratings (pain rating at *T*_120_ minus *T*_60_) [9]. This enabled us to obtain a delta score, which was compared to both groups. Peak pain was calculated by obtaining the mean pain score during this period (first 15 seconds of constant temperature stimulation). A *P* value of less than 0.05 was considered statistically significant.

## 3. Results

### 3.1. Sex Hormones Level

The mean values for each sex hormone are illustrated in Table 1. All subjects had sex hormones within normal levels for each phase according to the reference values obtained from the biochemistry laboratory at the *Centre Hospitalier Universitaire de Sherbrooke* (http://www.lab.chus.qc.ca/). Sex hormones were comparable for both groups, except for FSH and LH, which were higher in the PMW group (a normal endocrine manifestation of menopause).

### 3.2. Pain Perception

#### 3.2.1. Heat Pain Threshold and Heat Pain Tolerance

Heat PTh and PTol, as measured by the thermode temperature (°C) at which a subject reports the onset of pain or tolerance, were similar for both groups (*P* = 0.46 and 0.29, resp.). When the elapsed time (sec) before reaching PTh was evaluated, the PMW took significantly longer to report the onset of pain (43.1 ± 7.69 seconds versus 36.3 ± 13.2 seconds; *P* = 0.05), indicating a trend towards higher PTh (sec) for PMW (see Table 2). Moreover, we found a significant correlation (*r* = 0.31; *P* = 0.02) between age and PTh (sec). No significant correlation was found between age and PTol, mean CPT pain intensity, or PTh (°C).

#### 3.2.2. Tonic Pain Perception

**(1) Cold Pressor Test**

For an identical cold pain stimulus, the PMW reported significantly more pain during the CPT, where the mean pain intensity was 74.3 ± 24.8 compared to 57.44 ± 25.76 for the RMW (*P* = 0.005).

**(2) Heat Pain Stimulus (Thermode)**

We found no significant group difference in the mean pain ratings during the heat pain test, where the mean intensity for the 120-second period was 60.75 ± 23.1 for the PMW and 69.4 ± 17.1 for the RMW (*P* = 0.14). This was expected since pain intensity was individually adjusted to a VAS of 50/100. Furthermore, the thermode temperature used to evoke a pain score of 50/100 was similar for both groups (*P* = 0.18).

#### 3.2.3. Peak Pain and Temporal Summation

**(1) Peak Pain Period**

The RMW reported significantly higher pain intensity during the peak pain period (first 15 seconds of constant stimulation) compared to the PMW, where the mean ratings for this period were 70.6 ± 15.3 for the RMW versus 57.44 ± 25.76 for the PMW (*P* = 0.03) (see Figure 1).

**(2) Temporal Summation**

Temporal summation did occur for both groups during the tonic heat pain test, where pain intensity at *T*_120_ was significantly higher than at *T*_60_ (all *P* < 0.05). Furthermore, comparisons of the delta scores (*T*_120_ minus *T*_60_) obtained for each group were also similar (*P* = 0.39), indicating that both groups had comparable temporal summation during the tonic heat pain procedure. Finally, pain ratings at the last point in time (*T*_120_) were not significantly different (*P* = 0.38) (see Figure 1).

#### 3.2.4. DNIC Analgesic Effect on Tonic Heat Pain

**Figure 2:**
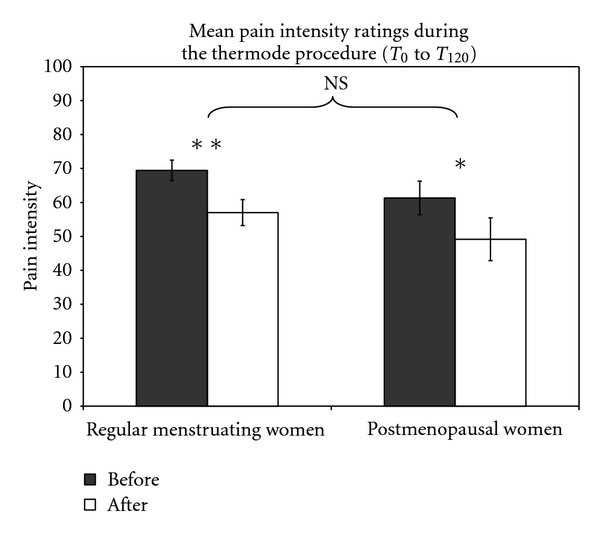
Mean heat pain ratings (thermode) were significantly lower after the CPT. Both groups had comparable changes (decrease) in pain intensity ratings following the CPT, indicating comparable DNIC analgesia.

Mean pain ratings for both groups were significantly lower during the thermode procedure following the CPT compared to the first tonic heat pain. This indicates similar DNIC analgesia for both the RMW and PMW (see Figure 2). This was also confirmed by comparing the delta scores (mean difference in heat pain perception before and after the CPT), which were similar for both groups (*P* = 0.99). Detailed results of the above sections (Section 3.2) are presented in Table 2. 

Finally, we reanalyzed the results for the variables where we found significant group differences (CPT pain, peak pain, and PTh) with covariance analysis controlling for FSH and LH (the only two sex hormones that significantly differed between both groups), only the “peak pain” remains significant (*P* = 0.02). 

## 4. Discussion

In this study, we examined pain responses to different experimental type of nociceptive stimuli in a group of young PMW and compared the results to nonmenopaused women. We decided to proceed with the experiment while the RMW were during their menstrual phase, with the rational being that sex hormones levels are more comparable to PMW [18]; blood sampling enabled us to assure that both groups had comparable sex hormones levels for PRO and EST, the main female sex hormone.

The main finding of this study is that the PMW showed an absence of peak pain and a trend towards a delayed PTh (sec) during the heat pain procedure as clearly illustrated in Figure 1. Since female sex hormones were comparable between both groups at time of testing, it suggests that age, not PRO or EST, is the main factor for these changes in nociceptive activity. However, since FSH and LH were higher in the PMW group, we cannot exclude a potential effect of the sex hormones. However, when we analyzed the results for the variables where we found significant group differences (CPT pain, peak pain, and PTh) and statistically controlled for FSH and LH, only peak pain remained significant. This further supports that the observed group difference in peak pain is probably due to age rather than sex hormones.

On a physiological perspective, we think that these changes in pain perception derive from decreased A*δ*-fiber function. Indeed, we and others have previously shown that the peak pain phase is always present during the tonic heat pain procedure; this has been demonstrated in young and middle-aged adults [9, 12, 14]. 

As Figure 1 clearly shows, the PMW took more time to reach their PTh (sec) but, most importantly, did not display the typical “rise and fall” pattern in pain perception observed during the first 15 seconds of constant stimulation. This difference in PTh (sec) is neither related to thermode temperature rate (both groups started at 32°C with a rising rate of 0.3°C/sec) nor to the thermode temperature used during the constant stimulation period (both groups had comparable temperatures). Therefore, an age-related factor would most likely account for these findings as suggested by the covariance analysis. Although a literature review concluded that there were no age-related changes in PTh or PTol [19], others previously demonstrated that aging produces an impairment in myelinated nociceptive fibers (i.e., A*δ* fibers) [20], that experimental PTh does increase with age [4, 21], and that C-fiber activity remains intact with aging [22]. Most importantly, Chakour et al. demonstrated a differential age-related change in A*δ* versus C-fiber pain perception. By blocking A*δ*-fiber function in a group of young (20–40 years) and older subjects (>65 years), they found that both groups had comparable C-fiber function. The thermal PTh (sec), however, was affected only in the younger group, suggesting decreased A*δ*-fiber function in the older subjects [20]. Although we found a significant correlation (*r* = 0.31; *P* = 0.02) between age and PTh (seconds), which indicated that only PTh increases with age, it only shows a modest association. 

Moreover, Tucker et al. [23] also reported a decreased A*δ*-fiber function as shown by an increased cutaneous pain threshold to the transcutaneous neuronal electrical stimulator. These findings closely relate to our study, since the peak pain period during the thermode test is most likely explained by A*δ*-fiber activity [13]. To our knowledge, there is very few relevant literature than these results and the other studies mentioned supporting such changes in nociception and aging [24].

We also found that endogenous pain inhibitory mechanisms, more specifically diffuse noxious inhibitory controls (DNICs), were also equivalent between both groups. This is somewhat different from what is found in the literature, since previous studies reported changes in DNIC with age [25, 26]. However, their samples were significantly older than our population of PMW. Furthermore, they compared their “older” groups to a group of young males and females, without controlling for menstrual-cycle phase, which is known to affect DNIC efficacy [27]. The effect of sex hormones on DNIC efficacy was significant only during the ovulatory phase, a phase which represents only 3 to 5 days of the complete menstrual cycle, while in the present study, the data were collected during the menstrual phase of the cycle [27]. 

A recent study conducted in our laboratories has also shown a decrease in DNIC efficacy after the age of 45 [2]. These results might be accounted for, in part, by the fact that a different experimental design was used and that sex hormones levels or menstrual-cycle phase was not controlled. Furthermore, although the PMW and RMW in our sample show comparable DNIC analgesia, the PMW reported more pain during the conditioning stimuli (cold pressor test) (see Section 3.2.2 (1)), yet they showed similar DNIC analgesia. This could suggest that the PMW would have had lower DNIC analgesia if they had lower pain score during the conditioning stimulus. However, we did not observe any significant interaction between DNIC efficacy (delta scores) and CPT pain intensity used as a covariable (*P* = 0.60). This observation strongly suggests that DNIC analgesia is therefore comparable in both groups. Consequently, we think that our PMW were probably too young to allow us to observe any age difference in DNIC. 

The results regarding tonic painful procedures and PTol suggest that PMW have comparable C-fiber nociceptive activity. Although our sample of PMW reported greater pain ratings during the CPT, we observed no significant difference in temporal summation of heat pain, an event mostly related to C-fibers [15]. The fact that PMW had higher pain intensity ratings during the CPT, but not elsewhere, might be related to the greater affective component of this test. The cold pressor test has been shown to induce higher estimates of unpleasantness, and thus may better mimic clinical pain [28], which 10 women in the PMW group reported. Furthermore, the fact that PMW had higher pain intensity ratings during the CPT could also be explained by the specific effect of hormonal changes on mood by menopause, where PMW are at higher risk of depression [29]; depression is known to negatively influence pain perception [30]. Also, since the cold pressor test pain is mainly related to the activity of C-fibers [31], it may well be a separate effect between a-delta and c-fibers. Finally, aging has previously been demonstrated to be related to a reduction of cutaneous pain but an augmentation of deep pain [32]. Since the thermode produces a cutaneous pain and that the cold pressor test produces deep pain, it may explain the apparently contradictory results.

These results have important clinical implications. First, it shows that quantitative sensory testing, such as the heat pain procedure, brings useful information for the detection of impairments in the peripheral nervous system. Moreover, since our test seems to discriminate A*δ* and C-fiber activity, it could serve as an objective criterion for measuring the severity of pain-related disorders, such as neuropathic pain.

Our study has potential limitations. First, we know that LH and FSH levels were higher in the PMW group. Hence, we cannot ignore the possibility that the observed differences in LH and FSH are indeed responsible in the observed pain responses rather than age. There is, however, no way of controlling for these two specific sex hormones. As mentioned in the introduction, the higher LH and FSH levels are natural manifestations of menopause. Moreover, past studies on pain perception and sex hormones did not reveal that LH or FSH had any effect on pain perception [18, 33]. Finally, the fact that 10 PMW reported lower-back pain could be a potential confounding factor. In fact, studies show that low back pain can sensitize [34] the central nervous system which could then explain why PMW women had greater pain during the cold pressor test. However, this could not explain why PMW had lower pain ratings (peak pain) during the 2-minute heat pain test with the thermode. However, since low back pain symptoms are frequent in the general population, it adds to the external validity, since the prevalence of painful conditions (such as low back pain) usually increases with age [4].

The age effects we observed are probably not limited to women. Since there was no control group of men, it would be imprudent to imply that the external validity of our results applies to men. More research is needed to address this question.

In conclusion, age seems to be the main factor influencing changes in tonic pain perception in our group of midlife PMW. The absence of the peak pain in the PMW was probably due to a reduction of function in myelinated A*δ* fibers that naturally occurs with age. Interestingly, these changes in pain perception occurred as early as 50 years old, which is congruent with recent literature [2]. These pain-related changes in postmenopausal women clearly demonstrate the importance of studying nociception and endogenous pain modulation in this population.

## Figures and Tables

**Table 1:** Sex hormones dosage by group.

	Sex Hormones: Mean ± SD (reference values)		
	RMW *n* = 32	PMW (*n* = 18)	*P* value*
Testosterone (total) (nmol/L)	1.15 ± 0.62 (0.7–2.8)	1.12 ± 0.57 (0.7–2.8)	0.86
Progesterone (nmol/L)	4.0 ± 4.1 (0.6–4.7)	2.51 ± 1.95 (0.3–2.5)	0.15
Estradiol (pmol/L)	151 ± 161 (46–607)	97.8 ± 66.0 (0–201)	0.18
FSH (IU/L)	6.15 ± 4.1 (3.5–12.5)	77.6 ± 36.4 (26–135)	<0.0001
LH (IU/L)	3.71 ± 1.19 (2.4–12.6)	40 ± 19.6 (8–59)	<0.0001

*RMW versus PMW.

Reference values for each hormone are reported in parenthesis.

**Table 2:** Group comparison of each pain measurement (mean value ± standard deviation).

Variable	RMW (*n* = 32) (Mean ± SD)	PMW (*n* = 18) (Mean ± SD)	*P* value
Pain threshold (°C)	42.5 ± 3.01	43.2 ± 3.84	0.46
Pain threshold (seconds)	36.35 ± 13.2	43.1 ± 7.69	**0.05**
Pain tolerance	47.07 ± 1.77	46.7 ± 2.49	0.29
Fixed thermode temperature (°C)	46.3 ± 1.0	45.64 ± 2.37	0.18
Peak pain (mean *T*_0_ to *T*_15_)	70.6 ± 15.3	57.44 ± 25.76	**0.03**
Last pain score (*T*_120_)	76.46 ± 19.76	70.66 ± 26.2	0.38
Thermode (before CPT) (mean *T*_0_ to *T*_120_)	69.4 ± 17.08	60.75 ± 23.1	0.14
Thermode (after CPT) (mean *T*_0_ to *T*_120_)	57.0 ± 21.6	48.89 ± 28.64	0.26
CPT mean pain intensity	53.8 ± 23.4	74.3 ± 24.8	**0.005**
TS delta score	9.57 ± 14.5	14.19 ± 17.34	0.39
DNIC delta score (*T*_120_ minus *T*_60_)	−12.4 ± 15.24	−11.85 ± 28.9	0.99
